# What do the differences and commonalities in doctoral dissertation acknowledgments across disciplines reveal?

**DOI:** 10.1371/journal.pone.0335035

**Published:** 2025-11-04

**Authors:** Kexin Yang, Jingwen Han, Huibin Zhuang

**Affiliations:** 1 School of Culture and Communication, Shandong University, Weihai, China; 2 International College of Chinese Studies, Shanghai Normal University, Shanghai, China; Universiti Malaya, MALAYSIA

## Abstract

Acknowledgments in academic dissertations occupy a unique role within scholarly communication. Prior research has investigated acknowledgments through lenses such as funding attribution, genre analysis, and linguistic features. This study examines acknowledgments in doctoral dissertations from Chinese universities, organized by broad disciplinary categories. Utilizing BERTopic modeling, the research identifies topic keywords embedded within dissertation acknowledgments. Furthermore, computational linguistics techniques are employed to quantitatively evaluate the content and stylistic attributes of these acknowledgments, complemented by hierarchical clustering analysis to explore cross-disciplinary similarities. The topic modeling results indicate that acknowledgments by Chinese doctoral students frequently convey emotional reflections and exhibit distinct disciplinary traits. Additionally, hierarchical clustering shows that disciplines with similar characteristics exhibit greater similarity in the content and writing style of their acknowledgments, indicating that academic training influences researchers’ writing to some degree. This study seeks to catalyze further scholarly inquiry into this domain, advocating for expanded investigations from perspectives including psychology, neuroscience, and cross-cultural studies.

## Introduction

Scientific research across most disciplines is rarely the product of solitary endeavor; rather, it typically necessitates the collective efforts of a research team and financial support. Contributions to scientific achievements are often reflected in the acknowledgments section of research articles. Previous studies on thesis acknowledgments have primarily focused on two aspects: quantitative analysis of funding support [1–5] and qualitative analysis of acknowledgment content. A recent study [6] analyzed funding entities in acknowledgment texts of articles published in English-language journals indexed in Web of Science (WoS) from 2014 to 2019, spanning social sciences, economics, oceanography, and computer science. The study found a positive correlation between the word count of acknowledgment texts and the number of acknowledged funding organizations, universities, individuals, and other entities.

Research on funding support primarily comes from journal articles, while studies on content have begun to focus on thesis acknowledgments, mainly through qualitative analysis. A pioneering study by Giles and Councill [7] was among the first to employ natural language processing for large-scale analysis of acknowledgment content to extract named entities. Similarly, Paul-Hus et al. [8] conducted a large-scale analysis of over one million research articles and reviews published in 2015, concluding that acknowledgments *“convey the indebtedness of authors to people and institutions whose contributions deserve to be publicly noted”* [8]. Paul-Hus and Desrochers [9] provided a qualitative analysis, which further explored acknowledgment content in scientific articles and reviews.

As research on acknowledgments has progressed, the focus has expanded beyond funding support to encompass discourse and linguistic analyses [12–15]. Researchers have increasingly recognized that acknowledgments in doctoral dissertations are richer in content compared to those in published articles. For instance, Mantai and Dowling [15] examined 79 doctoral dissertation acknowledgments from various disciplines across Australian universities, adopting a perspective centered on researchers’ social networks and relationships. Their findings indicate that doctoral candidates place greater emphasis on acknowledging social support—defined as emotional, moral, collegial, and guidance support—than academic or instrumental support.

Another research perspective examines dissertation acknowledgments through the lens of genre and rhetorical analysis, focusing on linguistic and socio-cultural dimensions [10,11,16]. Notably, several studies have identified the presence of religious elements in dissertation acknowledgments. Hyland (and his colleague Tse) [12–14] proposed a move structure framework for analyzing the rhetorical organization of acknowledgments in graduate dissertations. Building on this framework, Al-Ali [10] analyzed acknowledgments from 100 doctoral dissertations written by native Arabic speakers, further refining the structural framework by incorporating socio-cultural components, particularly religious elements. A distinctive feature in Arabic acknowledgments is the inclusion of expressions praising and thanking Allah, as well as invoking divine blessings. Similarly, Hyland [12] observed that scholars from various countries include rich and diverse content in their dissertation acknowledgments, with religious expressions such as “thanking God” frequently appearing:

As highlighted by Mantai and Dowling [15], acknowledgments in doctoral dissertations exhibit significantly richer content compared to those in published journal articles. Beyond expressing gratitude for funding support, doctoral acknowledgments often include appreciation for social support, making them notably more comprehensive in scope. This indicates that analyzing the content of thesis acknowledgments holds significant research value. However, previous studies have primarily relied on qualitative analysis, with less emphasis on quantitative or natural language processing (NLP) methods. In fact, topic modeling can efficiently extract key information from large volumes of content. For instance, An et al. [16] utilized topic modeling to analyze topics in thesis acknowledgments, identifying differences between co-national networks and acknowledgment networks. Similarly, Griffiths and Steyvers [17] developed a modeling algorithm to analyze abstracts in the PNAS. Through topic modeling, they uncovered meaningful aspects of scientific structures and revealed relationships between scientific papers across different disciplines, facilitating a better understanding of the information contained within vast knowledge domains.

Hannigan et al. [18] advocate for the application of topic modeling in management research. They highlight its practicality in constructing corpora, its ability to integrate with various quantitative and qualitative methods, and its applicability across diverse theoretical approaches as the foundation for its significant potential and promise in management studies.

These studies demonstrate the high feasibility and practicality of topic modeling in analyzing related issues. Therefore, we employ topic modeling to study acknowledgments in doctoral dissertations, with a focus on those in Chinese doctoral dissertations. This is because we have observed that when analyzing the acknowledgments in Chinese doctoral dissertations using relevant theoretical frameworks, distinct characteristics emerge. Chinese students’ dissertation acknowledgments often reflect on their doctoral journey, frequently highlighting the challenges encountered during their studies, such as the loss of loved ones. For instance, one acknowledgment from a 2021 economics doctoral dissertation at Jiangxi University of Finance and Economics states:

天有不测风云，人有旦夕祸福。读博第二年父亲因病去世，使我不但不能专心注重学习，而且要承担父亲遗留下的照顾瘫痪 10 年母亲的这份责任。（匿名作者 江西财经大学 经济学2021博士论文致谢 )

*Misfortunes come unexpectedly, as humans are subject to sudden calamities. In the second year of my doctoral program, my father passed away due to illness, which not only prevented me from focusing on my studies but also burdened me with the responsibility of caring for my mother, who has been paralyzed for ten years. (Anonymous author, Jiangxi University of Finance and Economics, 2021).*

Notably, these acknowledgments often incorporate classical Chinese poetry to express personal sentiments, while religious content is rarely included. For example, an acknowledgment from a 2021 education doctoral dissertation at Northeast Normal University begins by quoting a line from a Song dynasty poem:

莫等闲，白了少年头，空悲切。——《满江红》岳飞

*Translation: “Do not waste time idly, lest youthful vigor fades in vain regret.”*

*—Yue Fei, Man Jiang Hong (Full River Red)*

However, the author does not elaborate on the specific emotions intended by this poetic reference. In contrast, an acknowledgment from a history doctoral dissertation at Jilin University provides an explanatory context for the quoted poetry:

《诗经》说”哀哀父母，生我劬劳。”多年求学在外，不仅让父母牵挂悬心，还给家里增加了经济压力。父母两鬓已霜，但仍为了我终日奔波操劳，从无半句怨言。

The Book of Songs (Shijing) states: “哀哀父母，生我劬劳。”

*Translation: “It’s very hard for parents to give birth and raise their children.”*

*For many years, studying away from home has caused my parents constant worry and added financial burdens to our family. Though their hair has turned gray, they continue to labor tirelessly for my sake without a single complaint.*

By providing such context, the author ensures that readers, even those unfamiliar with the quoted poetry, can understand the intended emotional expression. Given these characteristics, the acknowledgments in Chinese doctoral dissertations resemble a literary genre closely intertwined with academic research. Prior studies have explored the relationships between academic style, cognitive processes, learning styles, and academic performance [19,20]. These studies indicate that disciplinary characteristics can influence researchers’ cognitive approaches. However, whether and how these influences manifest in non-academic writing, such as doctoral dissertation acknowledgments, remains underexplored in existing research.

In summary, the acknowledgments in Chinese doctoral dissertations hold significant research value, as they address several gaps in previous studies:

- In terms of research methods, prior studies have rarely employed quantitative analysis to examine the content of dissertation acknowledgments, with quantitative research primarily focusing on funding support.
- In terms of research materials, previous qualitative studies on dissertation acknowledgments have mainly targeted regions outside mainland China and have not examined the subject from a multidisciplinary perspective.

In fact, numerous scholars have explored the differences between humanities and social sciences and natural sciences, as well as the cognitive distinctions between researchers in these fields [21–25]. We believe that whether these differences influence the writing style of dissertation acknowledgments is a question of significant research value. Therefore, to enrich related research, this study utilizes topic modeling and quantitative analysis to examine the following research questions:

Q1: What are the primary topics in the acknowledgments of Chinese doctoral dissertations, and what insights do these topics provide?
Q2: Does the research disciplines of doctoral students influence the writing style of their dissertation acknowledgments?

In the following sections, we will utilize a self-constructed database and quantitative analysis to address the two research questions outlined above. It is important to clarify that this study excludes acknowledgments from master’s theses. This decision is informed by Hyland [12], who notes:

*Master’s students, on the other hand, typically study on a part-time basis and are looking forward to returning to their professional workplaces. Their theses tend to be much shorter (averaging only a third of the length of Ph.D.s), constructed fairly quickly, and completed in addition to substantial coursework. Not surprisingly, therefore, acknowledgments were afforded less significance by these master’s students, and some saw acknowledgments as merely a convention ([11]:p.249).*

We contend that the situation of master’s students in China aligns with Hyland’s observations. Furthermore, pursuing a doctoral degree in China is significantly more demanding than obtaining a master’s degree. Doctoral dissertation acknowledgments are thus more likely to reflect profound insights into the research process and the disciplinary field. In China, only programs at highly ranked institutions in specific disciplines are granted the authority to confer doctoral degrees. These institutions impose stringent requirements on doctoral candidates, such as the publication of multiple core journal articles as a prerequisite for graduation. We argue that the rigorous demands placed on doctoral students—reflecting the substantial effort required to earn the degree—result in acknowledgments that convey deeper reflections and insights.

## Data and methods

### Data collection

To investigate the commonalities and differences in acknowledgments of Chinese doctoral dissertations, this study constructed a small-scale database comprising dissertation acknowledgments. The database includes 360 acknowledgment sections from doctoral dissertations, with 30 randomly selected samples drawn from each of the 12 major disciplinary categories defined by Chinese higher education. The following points provide further clarification:

All acknowledgment sections were sourced from publicly available data on CNKI (https://www.cnki.net/) for dissertations completed between 2015 and 2021. CNKI is the largest national repository for Mainland China dissertations with standardized metadata and broad institutional coverage. Prior to 2021, authors and their institutions had the discretion to decide whether to upload and make public the acknowledgment sections of doctoral dissertations. As these sections may contain personal information (e.g., names of family or friends), uploading them implies the author’s consent to public disclosure. Starting in 2022, dissertations uploaded to CNKI no longer include acknowledgment sections.

The database was constructed using publicly available CNKI data from 2015 to 2021. For disciplinary classification, this study adopts the *Discipline Catalogue for Degree Conferral and Talent Cultivation (2018 revised edition)* issued by the Chinese Ministry of Education, which categorizes disciplines into 13 major fields: Philosophy, Economics, Law, Education, Literature, History, Science, Engineering, Agriculture, Medicine, Military Science, Management, and Arts. Due to the limited availability of publicly accessible dissertations in Military Science, this field was excluded from the study, resulting in the inclusion of 30 acknowledgment sections from each of the remaining 12 disciplinary categories.

It should be further noted that the number of dissertations varies across disciplines in the CNKI database. During data collection, we observed that some disciplines, such as History, have significantly fewer doctoral dissertations compared to others, such as Engineering. This discrepancy is attributed to the structure of disciplinary programs in higher education, where natural sciences have notably more doctoral degree-granting institutions than humanities and social sciences. We considered this variation but chose to extract an equal number of samples from each discipline. This decision was made for two primary reasons. First, this study conducts topic modeling on the overall acknowledgments of doctoral dissertations. If sampling were based on disciplinary proportions, acknowledgments from science and engineering fields would significantly outnumber those from humanities and social sciences. Topic modeling is sensitive to data volume, and if data is collected based on disciplinary proportions, it may lead to topics more aligned with science and engineering, thereby overlooking some topics in the humanities and social sciences. Second, the study later employs hierarchical clustering to quantify the similarity of acknowledgment sections across disciplines, which requires a balanced dataset across disciplines. Therefore, extracting an equal number of acknowledgments from each discipline better aligns with the research objectives. We believe this approach does not introduce potential bias to the study results due to the uneven distribution of acknowledgment sections across disciplines in the CNKI database.

Additionally, to ensure representativeness, the selection of dissertations avoided interdisciplinary fields with significant overlap. For each discipline, the most representative subfields were chosen. For instance, interdisciplinary programs such as “Management Science and Engineering,” which can be classified under both Management and Engineering in the *Discipline Catalogue for Degree Conferral and Talent Cultivation (2018 revised edition)*, were excluded. Similarly, for the History discipline, only dissertations explicitly categorized on CNKI under core subfields such as “Archaeology,” “Chinese History,” or “World History” were selected, as studies in intellectual history or literary history could be classified under Philosophy or Literature.

### Research methods

This study employs two primary methods to analyze acknowledgment texts: topic modeling and hierarchical clustering.

For topic modeling, we utilize BERTopic [26], which is a topic modeling tool based on natural language processing (NLP), designed to uncover hidden topics from large volumes of text data. It combines the strengths of deep learning and traditional machine learning, leveraging a language model called BERT to understand the meaning of text and then clustering similar texts into topics.

BERTopic is widely used in topic modeling research based on large-scale corpora [27,28]. In the study [27], researchers used Twitter posts as research material to compare four topic modeling methods: LDA, NMF, Top2Vec, and BERTopic. Based on human expert evaluations, NMF and BERTopic were found to perform the best. This is because BERTopic offers two significant advantages over traditional Latent Dirichlet Allocation (LDA) methods. First, BERTopic leverages the BERT model to generate semantically rich document and word embeddings, effectively capturing the contextual meanings of words and producing coherent and intuitive topic representations. In contrast, LDA relies on a bag-of-words model, which struggles to handle polysemy and complex semantics. Second, BERTopic employs HDBSCAN to automatically determine the number of topics, offering greater adaptability compared to LDA, which requires pre-specifying the number of topics. By applying BERTopic, this study can scientifically, efficiently, and intuitively identify the primary topics in dissertation acknowledgments.

We conducted a comprehensive analysis of acknowledgments from doctoral dissertations across 12 disciplines: Philosophy, Economics, Law, Education, Literature, History, Science, Engineering, Agriculture, Medicine, Management, and Art. A total of 360 acknowledgment texts were collected, with 30 samples from each discipline. Initially, the texts were segmented using the Python-based Jieba library for Chinese word tokenization. Subsequently, a stop-word removal process was applied to eliminate punctuation, function words, adverbs, adjectives, and other terms irrelevant to thematic content. Next, each acknowledgment text was vectorized. For this purpose, we employed the open-source acge_text_embedding model from Hugging Face (https://huggingface.co/aspire/acge_text_embedding), which was pre-trained on Chinese corpora and is capable of effectively capturing semantic information from Chinese texts.

For hierarchical clustering, we adopt two approaches:

Vector-based Clustering: Vectorized semantic representations obtained using word embedding techniques can effectively preserve the semantic content of text. As early as 2014, Baroni et al. [29] used a corpus of 2.8 billion tokens in the general English domain to systematically evaluate word vectors generated by four models. These models were tested on 14 benchmark datasets, demonstrating that text vectorization can effectively represent the semantic meaning of text. Therefore, we vectorize each acknowledgment text individually using a language model. For each disciplinary category, the average vector of all acknowledgment texts is computed, and these vector representations are used for hierarchical clustering.

Text Feature-Based Clustering: In addition to vectorizing text and performing hierarchical clustering, we also employed text readability metrics as text features for clustering. This is because, while text vectorization effectively preserves semantic information, high-dimensional vectors are not interpretable. Therefore, we introduced text readability metrics as feature parameters for clustering.

We analyze the text feature of acknowledgments by assessing the readability of Chinese texts, as literary sophistication in Chinese often correlates with reduced readability. This is due to the frequent use of idioms, historical allusions, and classical poetry, which enhance cultural depth and artistic expression but may obscure meaning for general readers due to their specialized, implicit, or context-dependent nature, thereby lowering readability. Lei et al. [30] developed AlphaReadabilityChinese, a tool for measuring the readability of Chinese texts, which evaluates readability across three dimensions—lexical richness, syntactic complexity, and semantic precision—using nine specific indicators. We apply these readability metrics to cluster acknowledgment texts from different disciplines, in order to enhance the reliability of clustering analysis from multiple perspectives.

## Results

### Primary topics in the acknowledgments of Chinese doctoral dissertations: Recollection, interpersonal relationships, discipline

At the beginning of the article, we proposed Q1: What are the primary topics in the acknowledgments of Chinese doctoral dissertations, and what insights do these topics provide? Topic modeling and analysis of associated topic words reveal three key characteristics of Chinese doctoral dissertation acknowledgments: recollection, interpersonal relationships, and discipline. Below is an introduction to the topic modeling and related analyses.

### Topic analysis of doctoral dissertation acknowledgments in China using BERTopic

BERTopic topic modeling involves parameter selection, requiring multiple attempts and adjustments to obtain the best-performing model. When evaluating model quality, some studies [31] use the overall “coherence” of topics as a standard for assessing model performance, calculating the semantic similarity of topic words directly or indirectly to ensure that topic words are not randomly combined but semantically related. Some studies also involve AI as a “judge” [32] or to assist in evaluating the quality of topic modeling [33]. Due to the specificity of the research object, topic modeling of doctoral dissertation acknowledgments may result in cases where a topic is related to a specific discipline, with topic words that are not semantically related but are all terms within that discipline. Therefore, in this study, we did not use the model evaluation methods from previous studies. Instead, we repeatedly adjusted parameters and manually analyzed and evaluated the modeling results.

**Fig 1:**
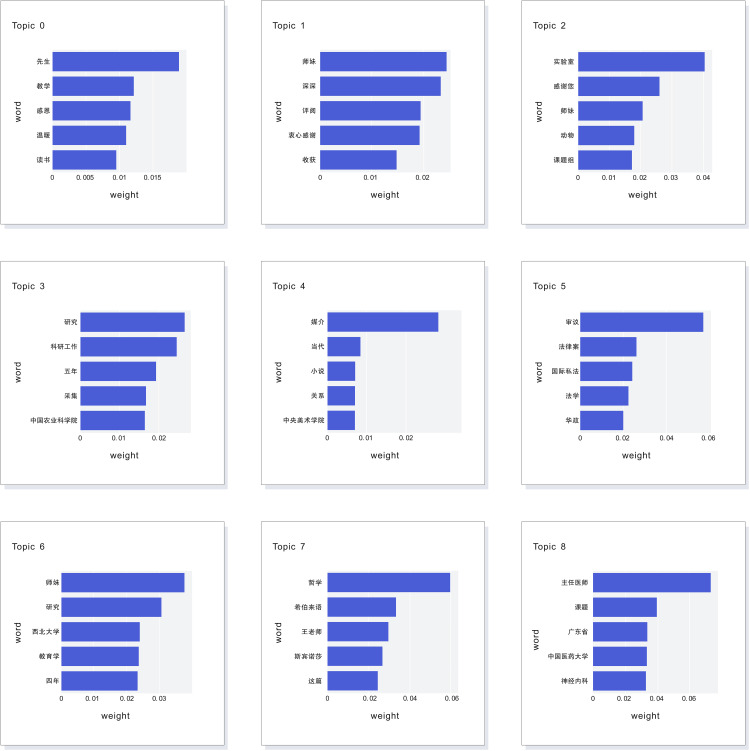
Visualization of the Top Five Topic Feature Words and Their Weights for Topics 0 to 8.

After extensive parameter tuning, optimal results were achieved by setting the HDBSCAN clustering model’s min_cluster_size to 10 and min_samples to 8, yielding the most coherent and interpretable topic clusters. Under these parameters, the BERTopic model identified nine core topics (Topic 0 to Topic 8). The visualization of the topic feature words and their corresponding weights (displaying only the top five feature words per topic by weight) is presented in Fig 1.

The topic words derived from the modeling analysis indicate that dissertation acknowledgments primarily encompass three dimensions: research focus, interpersonal relationships, and expressions of gratitude. The nine identified topics exhibit both commonalities and discipline-specific characteristics:

Firstly, many topics reflect distinct disciplinary traits. For instance, keywords in “Topic4” such as “当代” (contemporary), “小说” (novel), “媒介” (media), and “关系” (relationship), and in “Topic5” such as “审议” (deliberation), “法律案” (legal case), “国际私法” (private international law), and “法学” (jurisprudence), as well as “哲学” (philosophy) and “斯宾诺莎” (Spinoza) in “Topic7,” and “主任医师” (chief physician) and “神经内科” (neurology) in “Topic8,” are interwoven within the topics. These keywords suggest that authors frequently reference their academic disciplines and research directions in their acknowledgments, often recalling significant events from their doctoral journey. This is closely tied to the lifestyle of Chinese doctoral students, who typically engage in full-time research (“全日制”), with academic pursuits constituting the central focus of their work and lives during this period. Consequently, whether expressing sentiments, recalling memorable events, or conveying gratitude, the content is imbued with distinct disciplinary characteristics.

Additionally, the keywords in “Topic0” to “Topic2” center around gratitude for interpersonal relationships. Terms such as “先生” (a respectful address for teachers), “师妹” (junior fellow student (female)), and “课题组” (research group) refer to the recipients of gratitude, while terms like “感恩” (gratitude), “温暖” (warmth), “深深” (deeply), “衷心感谢” (heartfelt thanks), and “收获” (gains) directly express the authors’ feelings of appreciation.

Notably, the keyword “评阅” (review) frequently appears in Chinese doctoral dissertation acknowledgments, reflecting gratitude toward anonymous reviewers and professors involved in the defense process, despite the authors’ lack of knowledge about their identities. Although anonymous reviewers may not see these acknowledgments, such expressions are commonplace and can be viewed as a form of ceremonial gratitude or a reflection of the authors’ scholarly decorum.

**Fig 2:**
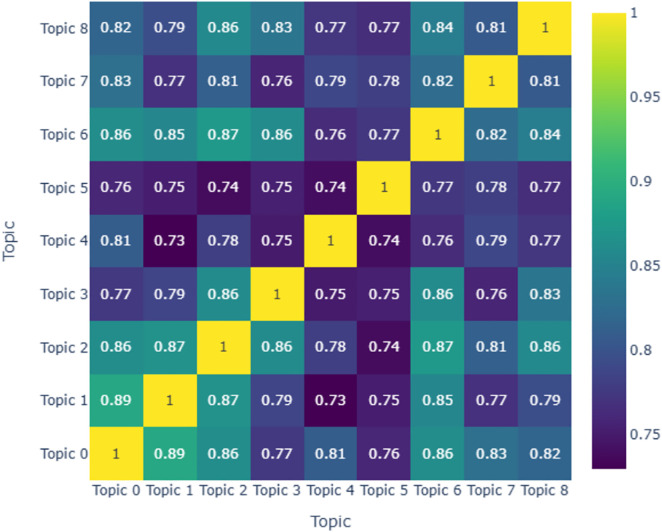
Heatmap of inter-topic similarity.

Furthermore, we calculated the similarity between different topics and visualized the results in the form of a heatmap, as shown in Fig 2.

The heatmap reveals variations in the similarity among the topics derived from the modeling process. Notably, higher similarity scores, ranging from 0.86 to 0.89, are observed between “Topic0” and “Topic1,” as well as between “Topic2” and “Topic6.” This may be attributed to these topics’ shared focus on expressions of gratitude. Conversely, lower similarity scores, ranging from 0.73 to 0.74, are found between “Topic1” and “Topic4,” “Topic2” and “Topic5,” and “Topic4” and “Topic5.” These findings suggest that dissertation acknowledgments, as a distinct genre, exhibit certain standardized expressions, such as gratitude toward supervisors or funding support, leading to some commonality across the identified topics. However, disciplinary differences also manifest, with science and engineering disciplines often acknowledging entire research groups, while humanities and social sciences disciplines tend to emphasize solitary textual research and greater interaction with supervisors rather than collaborative project completion.

In summary, based on the topic modeling, topic keywords, and analysis of 360 doctoral dissertation acknowledgments, we identify three key characteristics of Chinese doctoral acknowledgments:

- Recollection of personal experiences during the doctoral journey accompanied by emotional reflections.
- A strong emphasis on expressing gratitude for interpersonal relationships.
- Content marked by pronounced disciplinary characteristics.

The above analysis provides a general overview of the 360 dissertation acknowledgments. In the following sections, we will further explore the specific characteristics of acknowledgments across different disciplinary categories.

### Hierarchical clustering analysis of doctoral acknowledgments across disciplines

At the beginning of the article, we proposed Q2: Does the research discipline of doctoral students influence the writing style of their dissertation acknowledgments? In this section, we employed hierarchical clustering and found that dissertation acknowledgments from similar disciplines (e.g., those with similar research methods) are grouped closely together in the clustering results.

Johns and Swales [34] argue that the structure of doctoral dissertations is significantly influenced by the disciplinary field or subfield. They note that dissertations in some natural sciences often adopt an article compilation format, whereas those in the social sciences or humanities typically follow a topic-oriented structure. Intrigued by this observation, we sought to investigate whether doctoral dissertation acknowledgments are similarly shaped by disciplinary influences. To this end, we first conducted a preliminary statistical analysis of the average word count of acknowledgments across different disciplines, as presented in the table below (Table 1):

**Table 1:** Average Word Count of Doctoral Dissertation Acknowledgments Across Disciplines.

Discipline	Education	Law	Literature	Philosophy	History	Science	Economics	Engineering	Agronomy	Management	Medicine	Art
**Average number of Chinese characters**	1544.43	1430.87	1103.77	1094.83	1069.83	951.57	950.3	943.47	884.9	881.5	836.33	824.83

From the perspective of word count, the top five disciplines with the highest average word counts for doctoral dissertation acknowledgments are Education, Law, Literature, Philosophy, and History. However, the differences in word count are minimal, and it cannot be inferred that the acknowledgment content in these disciplines is more similar based solely on length. For instance, while both poetry and notices may be concise, the former is characterized by literary qualities, whereas the latter is straightforward and direct. Therefore, to further investigate the similarities, we employed vectorization and readability analysis to conduct hierarchical clustering.

### Hierarchical clustering results of vectorized acknowledgment texts

**Fig 3:**
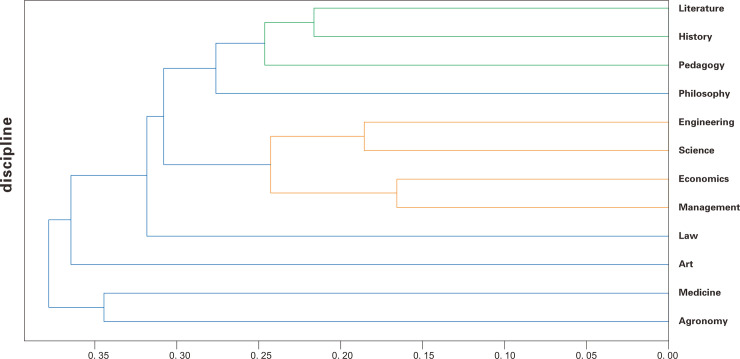
Results of Hierarchical Clustering of Vectorized Doctoral Dissertation Acknowledgments.

Text vectorization is a fundamental technique in natural language processing, as it effectively preserves the semantic content of text while facilitating efficient mathematical computations. By transforming text into high-dimensional vector representations, such as word embeddings or sentence embeddings (in this study, the “aspire/acge_text_embedding” model was used for sentence embedding), the intricate relationships among vocabulary, syntax, and semantics can be captured, thereby retaining the text’s semantic information. Moreover, vectorized data adopts a standardized numerical format, which simplifies tasks such as similarity computation, classification, and clustering. In this study, each doctoral dissertation acknowledgment underwent Chinese word segmentation and stop-word removal. The pre-trained “aspire/acge_text_embedding” model was then employed to convert the processed texts into vector representations. Subsequently, the average vector for all acknowledgment texts within each disciplinary category was calculated. Hierarchical clustering was performed on these categories using the Unweighted Pair Group Method with Arithmetic Mean (UPGMA) as the clustering algorithm, with the average distance between clusters computed. The Euclidean distance metric was applied to measure differences between the embedded vectors, resulting in the hierarchical clustering dendrogram presented below (Fig 3):

Hierarchical clustering analysis reveals that Literature, History, and Education form a closely related cluster. These disciplines are typically classified as humanities. Researchers in these fields often engage extensively with a large volume of literary texts and archival documents. Engineering and Science also cluster closely, reflecting their emphasis on rigorous computation. Similarly, Medicine and Agriculture form a tight cluster, likely due to their shared reliance on extensive experimental procedures. Management and Economics constitute another cluster, consistent with their mutual focus on commercial and financial systems.

The clustering results, derived from vectorizing textual semantic information using a language model, reveal significant internal differences in acknowledgment content across disciplines, which can be broadly categorized into four groups:

Humanities disciplines that emphasize textual analysis and critical reflection;

Social Sciences disciplines that focus on societal and commercial systems;

Science disciplines centered on rigorous computation;

Engineering disciplines that prioritize practical operations.

Furthermore, the analysis reveals considerable similarity in doctoral dissertation acknowledgments within each of these disciplinary categories. This pattern suggests that disciplinary specialization influences researchers, at least in the composition of their dissertation acknowledgments, resulting in a correlation between the similarity of acknowledgment content and the degree of disciplinary proximity.

### Results of hierarchical clustering based on acknowledgment readability data

Beyond the influence of disciplinary specialization on acknowledgment content, we also examined whether disciplines shape researchers’ writing styles through clustering based on readability metrics.

**Fig 4:**
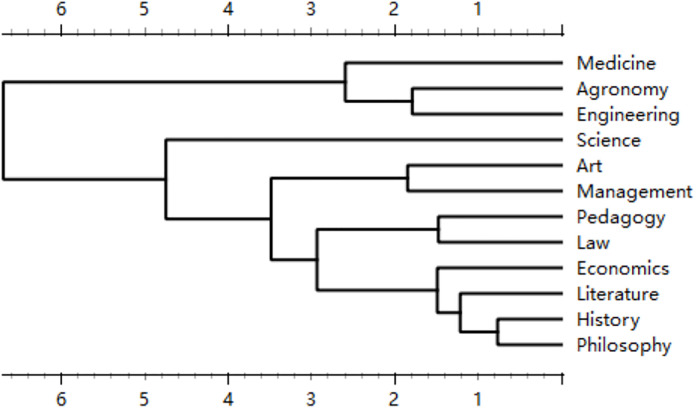
Results of Hierarchical Clustering of Doctoral Dissertation Acknowledgments Based on Readability.

Lei et al. [30] proposed a framework for analyzing text readability based on metrics such as Lexical Richness, Syntactic Richness, Noun Semantic Accuracy, Verb Semantic Accuracy, Noun and Verb Semantic Accuracy, Content Word Semantic Accuracy, Noun Semantic Richness, Noun Semantic Clarity, and Noun Semantic Noise. They posited that text readability can quantitatively reflect textual characteristics. In their study, Lei et al. [30] utilized works by Chinese martial arts novelists Jin Yong and Gu Long as source materials, demonstrating that nine readability metrics derived from a Chinese text readability tool effectively clustered the authors’ works into two distinct categories. They further suggested that these metrics exhibit high applicability in fields such as authorship identification. We contend that Chinese text readability analysis tools can substantially capture statistical features of texts. Moreover, employing readability to differentiate the literary quality of texts is highly relevant for Chinese texts, as authors with strong literary competence often incorporate allusions, idioms, and poetic expressions, which tend to reduce readability. Accordingly, this study calculates the average readability values of acknowledgments in doctoral dissertations across various disciplinary categories, using these values as the basis for hierarchical clustering. The results are presented in Fig 4.

Notably, the disciplines of Literature, History, and Philosophy form a closely related cluster, aligning with the traditional Chinese notion that “Literature, History, and Philosophy are inseparable”(”文史哲不分家”). Economics is the closest discipline to this cluster, likely due to its research often involving political, cultural, and policy-related studies, which share significant overlap with the reading materials of these disciplines. This similarity may, to some extent, influence researchers’ writing styles.

Agriculture and Engineering form another closely related cluster, possibly due to their shared reliance on extensive experimentation. Medicine follows closely, as medical research also involves substantial experimental work. This likely influences scholars’ writing styles, characterized by concise and focused articulation of key points, akin to writing experimental reports.

Overall, the 12 disciplinary categories are grouped into three clusters: natural science and technology disciplines (e.g., Agriculture, Engineering, Medicine); typical humanities disciplines (e.g., Literature, History, Philosophy); and management-oriented disciplines (e.g., Economics, Law, Management). During their doctoral studies, researchers extensively engage with discipline-specific materials (e.g., experimental reports, academic papers, books), which they use to produce scholarly writing. The clustering results suggest that closely related disciplines share significant commonalities. It appears that immersion in a specific discipline influences researchers’ cognitive and writing styles, as evidenced by the distinct disciplinary characteristics observed in their acknowledgment texts.

## Conclusion

Hyland [12] points out that dissertation acknowledgments primarily focus on six aspects: academic support, access to data, moral support, clerical services, financial resources, and technical help. From our thematic modeling study in the preceding text, we found that while Chinese doctoral dissertation acknowledgments also encompass these aspects, they distinctively include a significant amount of emotional expression—sometimes to the extent that they can hardly be considered “thanks.” For instance, some acknowledgments begin by reflecting on the passage of time, often starting with phrases like “Time flies by, the passing years never come again” as the opening of the acknowledgment section. Some dissertations even incorporate the authors’ personal memories of campus or laboratory experiences, which are difficult to categorize as “thanks” and can only be described as “reflections.”

We argue that this characteristic of Chinese doctoral dissertation acknowledgments does not deviate from the theme of “thanks” but rather constructs a unique significance for the doctoral degree through reflective expressions. Existentialist philosopher Jean-Paul Sartre believes that man exists and must create his essence and values for himself because he has freedom [35]. This perspective suggests that significant events (obtaining a doctoral degree can be considered a significant event) compel individuals to confront the absurdity of existence, thereby constructing personal meaning through reflection and action (such as emotional expression). This construction of personal meaning is, in fact, an acknowledgment of oneself—thanking one’s own efforts for achieving a highly meaningful doctoral degree. We believe that in the Chinese cultural context, attributing achievements to others rather than oneself is considered a humble and refined gesture. Therefore, when Chinese scholars obtain their doctoral degrees and write acknowledgments, they do not directly thank themselves but are more inclined to express reflections.

From our hierarchical clustering analysis in the preceding text, we found that the similarity in acknowledgments across different disciplines correlates with the degree of similarity between disciplines. Overall, there is a significant difference between natural sciences and humanities. Some scholars have discussed the differences between these two types of disciplines. A key distinction between humanities and natural sciences, as articulated by Zhou ([22]: p 243), is that “*people could not put related questions into calculation, as what they do in sciences, for example by mathematical formulas or experiments, when involved in theory disputation. In other words, there is no such authoritative common methodology program in the humanities as in sciences. Experiment is the soul of scientific methods, and the lack of uniform methods in the humanities ultimately results from the lack of the process of experimental demonstration.*”

This fundamental difference, rooted in disputational rather than mathematical reasoning, reflects variations in learning approaches, academic habits, and cognitive frameworks. These differences manifest in the acknowledgments of academic dissertations. Our analysis reveals that, despite commonalities across Chinese doctoral dissertation acknowledgments, vectorization of textual content and readability analysis through hierarchical clustering highlight clear disciplinary similarities. Acknowledgments from similar disciplines exhibit greater resemblance in both content and writing style.

These findings address the question raised at the outset of the study: topic modeling of dissertation acknowledgments elucidates the characteristics of Chinese doctoral acknowledgments, while hierarchical clustering reveals that disciplinary characteristics influence researchers’ acknowledgment writing, with this influence converging based on disciplinary similarity.

While this study extensively demonstrates the impact of disciplinary specialization on researchers’ cognitive frameworks and writing styles, it relies on dissertation acknowledgments as its primary material. As a product of cognitive processes, acknowledgments allow us to infer the influence of disciplinary study on scholars’ thinking only indirectly. Additionally, to highlight the specificity of Chinese doctoral dissertations, this study exclusively analyzed acknowledgment texts from mainland China. This topic holds significant potential for further exploration. We hope that future research, inspired by this study, will adopt interdisciplinary approaches—drawing on psychology, neuroscience, and cross-cultural communication—to more comprehensively investigate the impact of disciplinary study on researchers’ cognitive frameworks.

## Supporting information

S1 Raw Images All figures collection. This PDF includes all supplementary figures from the study, showing detailed experiment results.
(PDF)
