# Supplementary figures and images for: What do the differences and commonalities in doctoral dissertation acknowledgments across disciplines reveal?
Source: PLoS One. 2025 Nov 4;20(11):e0335035. doi: 10.1371/journal.pone.0335035 (PMC12585057; doi:10.1371/journal.pone.0335035)

Fig1

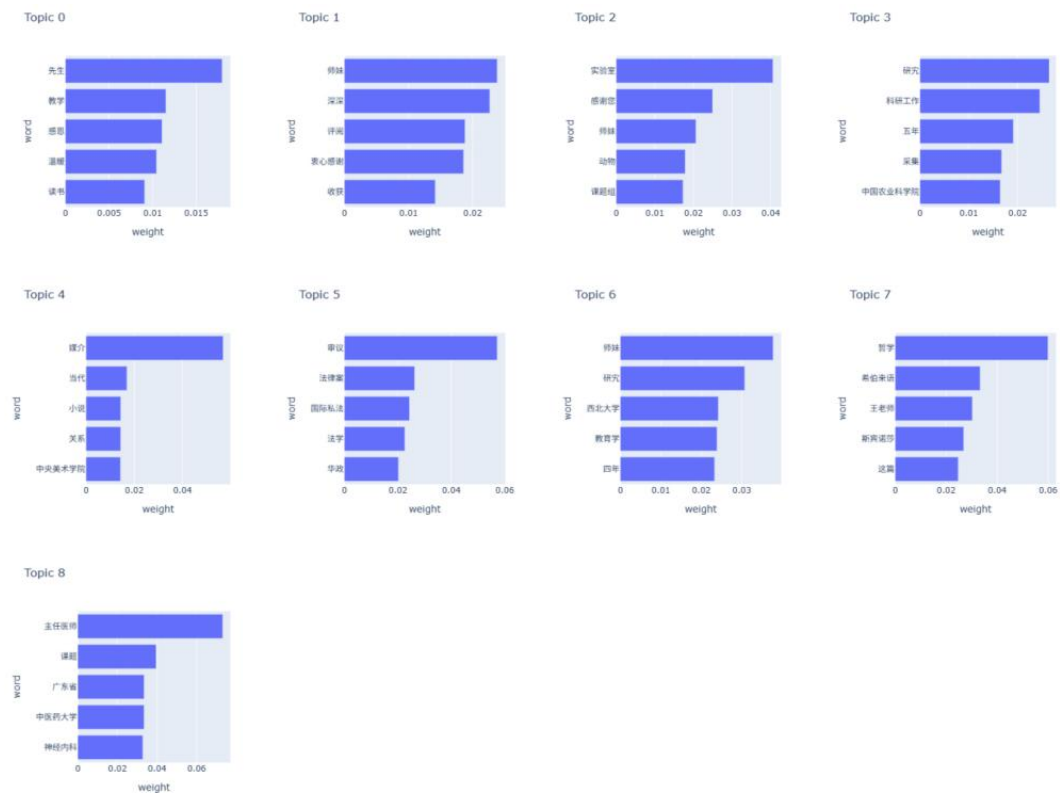

Fig2

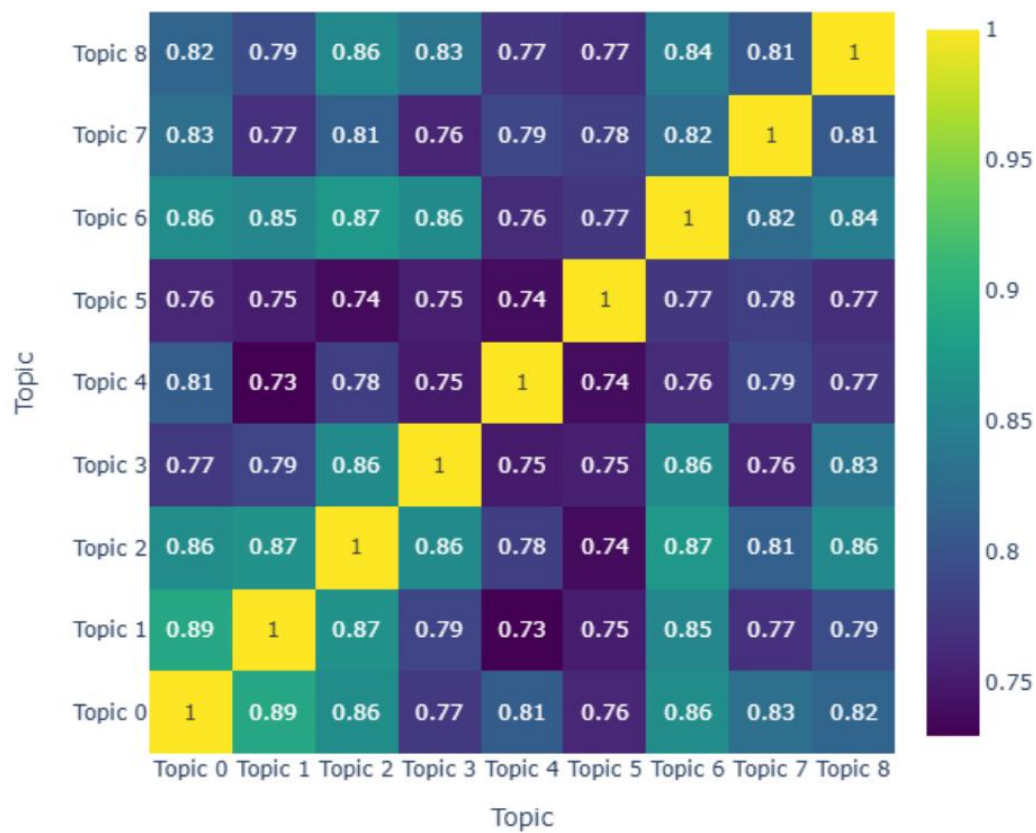

Fig3

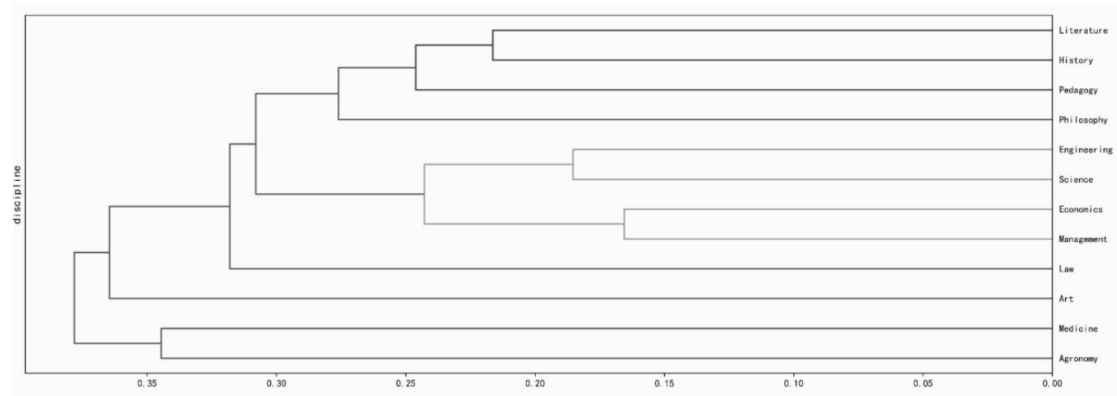

Fig4

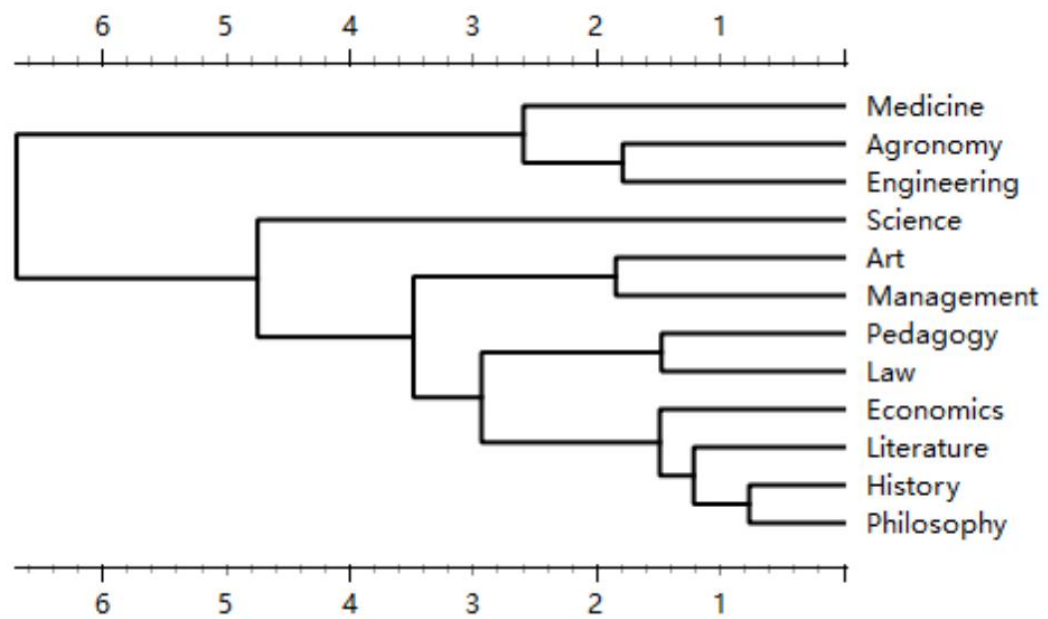

Supplement: S1 Raw Images — This PDF includes all supplementary figures from the study, showing detailed experiment results. (PDF) [file pone.0335035.s001.pdf]
